# Self-care activities in pediatric patients with type 1 diabetes mellitus

**DOI:** 10.1371/journal.pone.0300055

**Published:** 2024-03-05

**Authors:** Mirjana Smudja, Tatjana Milenković, Ivana Minaković, Vera Zdravković, Jovan Javorac, Dragana Milutinović

**Affiliations:** 1 Department of Higher Medical School, Academy for Applied Studies Belgrade, Belgrade, Serbia; 2 Faculty of Medicine, University of Novi Sad, Novi Sad, Serbia; 3 Department of Endocrinology, Mother and Child Health Care Institute of Serbia "Dr Vukan Čupić", Belgrade, Serbia; 4 Health Center Novi Sad, Novi Sad, Serbia; 5 University Children’s Hospital Belgrade, Belgrade, Serbia; 6 Faculty of Medicine, University of Belgrade, Belgrade, Serbia; 7 Institute for Pulmonary Diseases of Vojvodina, Sremska Kamenica, Serbia; 8 Department of Nursing, Faculty of Medicine, University of Novi Sad, Novi Sad, Serbia; Instituto Mexicano del Seguro Social, MEXICO

## Abstract

**Introduction:**

Effective diabetes self-management and collaborative responsibility sharing with parents are imperative for pediatric patients with type 1 diabetes mellitus, particularly as they gradually assume more self-care responsibilities. The primary goal of this study was to assess differences in adherence to self-care activities regarding sociodemographics and clinical characteristics in pediatric patients with type 1 diabetes. The secondary goal of this study was to understand the level of parental involvement in diabetes management and to assess the pediatric patients’ behaviors (independent or dependent on disease self-management) that relate to sociodemographic and clinical characteristics.

**Methods:**

This was a comparative cross-sectional and correlational study. The study sample included 182 children and adolescents who had been diagnosed with type 1 diabetes at least 3 months prior. Data collection instruments included a sociodemographic and questionnaire about Adherence to self-care activities and parental involvement in diabetes self-management, as well as a documentation sheet for recording clinical data.

**Results:**

A majority of participants (71%) exhibited non-adherence to self-care tasks, despite 78.0% asserting their independence in diabetes self-management. Notably, insufficient parental involvement in administering insulin therapy significantly predicted severe hypoglycemic episodes.

**Conclusions:**

Pediatric patients dealing with type 1 diabetes demonstrate a substantial degree of autonomy in managing their condition, paradoxically coupled with self-reported non-adherence to critical self-care responsibilities. Notably, children (aged 8–12) rely more heavily on parental support, especially concerning insulin therapy administration. The study underscores the crucial role of parental engagement in insulin therapy, as its deficiency significantly predicts the likelihood of severe hypoglycemic episodes.

## Introduction

Type 1 diabetes mellitus (T1DM) is typically diagnosed in pediatric patients. The International Diabetes Federation has projected that the global count of children and adolescents (aged 0–19 years) with T1DM was around 1.2 million in 2021, and approximately 184,100 new cases are identified each year [1]. Moreover, T1DM stands as one of the prevalent non-infectious chronic conditions among Serbian youths (aged 0–19 years), displaying a substantial incidence rate of 16.4 per 100,000 individuals. Examination of the National Diabetes Registry of the Republic of Serbia (Serbia) has unveiled a considerable yearly surge in recently diagnosed pediatric patients with T1DM, notably in the age groups of 5–9 (17.1 per 100,000 individuals) and 10–14 years (29.2 per 100,000 individuals) [2].

Following the identification of newly developed T1DM in children of school age, there are shifts in the child’s daily schedule and the entire family dynamic. Throughout this period, the essential factor becomes parental backing and engagement, as it plays a crucial role in motivating patients to independently manage their care routines and acclimate to the challenges brought about by this demanding scenario. Consequently, the enthusiasm of both the young patients and their parents to embrace and carry out the essential self-care tasks holds immense significance in shaping both short- and long-term health outcomes [3]. There is little data available from developing countries on pediatric patients’ self-care activities, and it seems that this concept has not been thoroughly investigated in children and adolescents outside developed countries.

Different definitions of self-care can be found in the literature that is currently accessible [4, 5]. Owing to the broad perspective of general well-being as opposed to narrow disease self-management and prevention, the concept of self-care was defined by Kickbush [6] as integrating three dimensions: psychosocial life, general health, and responses to illness demands. From the perspective of health promotion, supporting self-care (either independently or with parental assistance) entails improving the general well-being and quality of life of children and adolescents with chronic illnesses. In pediatric settings, where children and adolescents with T1DM have complex medical and developmental needs, this is especially important [4]. Within the framework of our study, “self-care can be defined as the execution of essential tasks concerning the well-being and health of children diagnosed with T1DM”. These tasks are carried out either autonomously by the pediatric patients themselves or with the assistance of their parents or guardians, particularly when the child’s capabilities are insufficient [4]. Chieng and colleagues [5] have deduced that the degree of self-care among pediatric T1DM patients is closely intertwined with their developmental stage and chronological age. Additionally, T1DM is linked with a multitude of risks and more than 600 intricate responsibilities that are imperative for the effective management of this chronic ailment [7]. These responsibilities encompass not only the physical undertakings crucial for maintaining glycemic levels but also the emotional adaptation to living with a chronic medical condition [8].

Regimen adherence encompasses the extent to which patients adhere to the guidelines provided by their healthcare practitioners [9]. In the context of pediatric T1DM patients, adhering to the regimen necessitates significant involvement from both the young individuals and their parents. This involvement encompasses various tasks, such as frequent glucose monitoring ranging from four to six times daily, making decisions regarding carbohydrate consumption, administering insulin injections, and adjusting therapy doses based on factors like physical activity, emotional state, and the presence of acute illness or infection [10]. Despite the advancements in diabetes treatment technology, evidence indicates that maintaining consistency in self-care routines remains a challenge for pediatric patients, with adolescents in particular facing difficulties [11]. This challenge can have repercussions on metabolic control. Therefore, healthcare professionals must consider all aspects of the daily routine of every pediatric patient and family member who is involved in diabetes management tasks. Especially considering that the pharmacokinetics of administered insulin and insulin sensitivity differ statistically significantly among patients. In addition, the amount of injected insulin that is absorbed may be highly variable even within individual patients [12, 13].

Previous research has emphasized the significance of self-management in the realm of children’s self-care, encompassing the adherence to daily routines and the collaborative sharing of responsibilities for diabetes-related tasks and decision-making with parents [14]. The process of transferring responsibility for self-care should be gradual and supervised. There is a time when 1) adults ((parents or guardians and doctors or diabetes specialist nurses (DSNs)) are fully responsible; 2) time when adults (parents or guardians and doctors or DSNs) prepare the child for responsibility, 3) time when adults ((parents or guardians)) monitor behavior, and 4) time when adults ((parents or guardians)) transfer full responsibility to the child [7]. Education and re-education programs are available to pediatric patients with diabetes and their parents or guardians in Serbia to provide adequate training for the self-management of diabetes [15]. When lacking adequate support from diverse sources like families, healthcare professionals, and peers at school, T1DM can profoundly disrupt the lives of pediatric patients, particularly adolescents who grapple with maintaining proper adherence to the T1DM regimen, often resulting in unstable blood glucose levels. Insufficient control of T1DM can lead to grave situations such as severe hypoglycemia episodes, diabetic ketoacidosis (DKA), and nonketotic hyperosmolar coma [16–19]. Moreover, it can hasten the progression of both microvascular and macrovascular complications, encompassing conditions like heart disease, neuropathy, nephropathy, retinopathy, and even premature mortality [7, 18]. Hence, a precise evaluation of adherence to self-care tasks holds paramount importance for effective healthcare delivery and for gauging both short- and long-term health outcomes. This chronic health condition places a heavy burden on the lives of pediatric patients, necessitating robust support from parents and families. The exploration of these experiences from the perspectives of children, adolescents with T1DM, and their parents has been the central focus of several investigations [20–23].

However, data on the experiences of self-care among Serbian children with T1DM are scarce. Therefore, the primary goal of this study was to assess differences in adherence to self-care activities (adherence to glycemic control and dietary regime, physical activity, and control of diabetes during school stay) regarding sociodemographics (age, gender, or region of residence) and clinical characteristics (episodes of severe hypoglycemia or DKA during the previous month) in pediatric patients with T1DM. The secondary goal of this study was to understand the level of parental involvement in diabetes management and to assess the pediatric patients’ behaviors (independent or dependent on disease self-management) that relate to sociodemographic (age, gender, or region of residence) and clinical characteristics ((glycosylated hemoglobin (HbA1c) level as an indicator of metabolic control, particularly with episodes of severe hypoglycemia or DKA during the previous month)).

Specifically, we tested two hypotheses:

Null hypothesis 1: There are no differences in adherence to self-care activities regarding sociodemographics (age, gender, or region of residence) or clinical characteristics (episodes of severe hypoglycemia or DKA during the previous month) in pediatric patients with T1DM.

Null hypothesis 2: There is no relationship between parental involvement in diabetes self-management and different sociodemographic (age, gender, or region of residence) or clinical characteristics (HbA1c level as an indicator of metabolic control, particularly with episodes of severe hypoglycemia or DKA in the previous month).

## Materials and methods

### Study design

The study took the form of a comparative cross-sectional and correlational investigation.

### Study population

A comprehensive convenience sampling strategy was employed, encompassing a total of N = 182 pediatric patients with T1DM in school-age range. These participants were categorized based on age into two distinct groups that included both genders: children (aged 8−12 years) and adolescents (aged 13−18 years).

The study inclusion criteria were: age 8–18 years, T1DM diagnosed at least 3 months prior, and absence of cognitive problems. Individuals with other types of diabetes and cognitive disabilities were excluded from the study. Data essential for achieving the study’s objectives were collected in written format during the period spanning 8 April 2020, to 30 December 2021, at the Mother and Child Health Care Institute of Serbia "Dr Vukan Čupić" and the University Children’s Hospital in Belgrade.

### Sample size calculation

The required sample size was calculated using the G*Power 3.1.3 software package for the following parameters: medium effect size w = 0.3; error α = 0.05; error β = 0.20; study power 80%; Df = 6. The minimum sample size was N = 159.

### Data collection

All pediatric patients (N =  182) who attended control check-ups or were hospitalized during the study period had equal opportunities to participate in the study (if they met the inclusion criteria and were willing to participate). Pediatric patients with T1DM and their parents/guardians were referred to the principal investigator’s room by pediatric endocrinologists who are co-authors of the report, as well as pediatric nurses from the admitting clinic.

In accordance with ethical protocols, every participant and/or their parents/guardians were provided written notification concerning the study’s objectives and the exclusive scientific use of gathered data. This assurance encompassed the preservation of data anonymity and the safeguarding of the identities of children and adolescents grappling with T1DM (only authors had access to information that could identify individual participants during or after data collection). Moreover, Informed consent (in writing) for participation in the research was obtained either from parents/guardians (for children and adolescents aged under 15), or directly from the pediatric patients (for those aged 16 and older), in accordance with the basic provisions of the “Law on patient rights” in the Republic of Serbia (“Official Gazette of the RS”, no. 45/2013 and 25/2019 –National Law, Article 2, paragraphs 4 and 5) [24]. These procedures included the completion of sociodemographic surveys as well as questionnaires relating to adherence to self-care activities and parental involvement in diabetes self-management. For this study, children and adolescents were not exposed to any additional diagnostic or painful procedures. Pediatric patients also had the option to refuse to answer any specific questions or to leave the study at any point, even after they or their parents (or guardians) signed the consent form. To ensure an appropriate atmosphere and privacy, pediatric patients were afforded separate rooms for completing the surveys. They were also asked to fill out the questionnaire independently (without parental help) and silently. Throughout this process, pediatric patients and their parents (or guardians) were together in a designated room, and one of the researchers remained on hand to address any queries or concerns. Аll the questionnaires were conducted at the same time altogether. Incomplete responses were not analyzed. Additionally, a documentation sheet for capturing clinical information was meticulously completed by the researcher.

### Measures

**Sociodemographic questionnaire** was used to obtain the following information: age, gender, a form of school attendance (full-time or part-time), school achievements (participants self-reported school achievement ranging from excellent to unsatisfactory), family structure (<5 or ≥5 members), and the geographical area of residence (encompassing all regions of Serbia: Belgrade, Vojvodina, Southern and eastern Serbia, Sumadija and western Serbia).

Note: In the context of this research, a family was delineated as "a unit of close individuals cohabiting with deep emotional connections (such as identification, attachment, loyalty, reciprocity, and solidarity), and with a shared history and future" [25].

**The documentation sheets sourced from the Heliant Health Information System electronic database** were employed to acquire the ensuing information: age of onset categorized into brackets of 0–4, 5–9, 10–14, and 15–18 years; the duration of T1DM classified as either <5 or ≥5 years; insulin administration method (insulin pen or insulin pump); glycosylated hemoglobin (HbA1c) levels utilized as an indicator of metabolic control, where HbA1c% values <5.7% (<38.8 mmol/mol) indicated ideal metabolic control, values ranging HbA1c 5.7% - 6.9% (38.8–51.9 mmol/mol) represented good metabolic control, values ranging HbA1c 7.0% - 8.5% (53.0–69.4 mmol/mol) indicated unstable metabolic control, and values HbA1c >8.5% (>69.4 mmol/mol) denoted poor metabolic control; instances of severe hypoglycemia and diabetic ketoacidosis (DKA) occurring in the preceding month were also collated.

Note: Severe hypoglycemia was categorized as a blood glucose level <2,2 mmol/mol (<40.00 mg/dL) with the presence of cognitive impairment such as seizures and severe hypoglycemic event resulting in loss of consciousness, convulsions, or coma [26].

Note: DKA episode was categorized as blood glucose level >11 mmol/L (>198mg/dL), venous pH <7.3 or serum bicarbonate <15 mmol/L (15mEq/L), and either presence of ketonemia ((blood β-hydroxybutyrate level ≥3 mmol/L (≥0,03 mg/dL)) or moderate to high ketonuria [27], which was an indication for hospitalization of the child/adolescent.

#### Adherence to self-care activities

Given the absence of Serbian assessment tools for evaluating self-care activities in children and adolescents with T1DM, as well as parental involvement in diabetes self-management, we employed a specially crafted questionnaire for our study. The initial segment of the questionnaire focused on “adherence to self-care activities” and comprised inquiries concerning four distinct behavioral aspects: adherence to glycemic control, dietary guidelines, physical activity, and challenges encountered while managing diabetes at school. The subsequent part of the questionnaire centered on assessing the extent of “parental involvement in diabetes self-management”, appraised through two key aspects: insulin therapy methods (such as multiple daily injections (MDI) or insulin pump therapy) and glycemic control approaches (including self-monitoring of blood glucose levels (SMBG) or continuous glucose monitoring (CGM) (S1 Appendix -Table format of the questionnaire). To ensure the questionnaire’s comprehensiveness and scoring system’s appropriateness, the research team meticulously reviewed published studies [28–31], thus facilitating comparability with other research findings. The questionnaire underwent content validity assessment, involving input from an endocrinology faculty member and two pediatric nurses. Additionally, face validity was evaluated by soliciting feedback from four parents and four pediatric patients (two aged 8–12 and two aged 13–18), who provided insights into the clarity, simplicity, and comprehensibility of the questions and response options.

*Description of the first part of questionnaire*. Evaluation of *glycemic control adherence* was determined based on responses to the subsequent questions, covering the preceding 30-day period: (1.) "How do you manage your glucose levels?" For respondents selecting SMBG, the following inquiries were posed: (2a.) "Do you require assistance when measuring capillary blood glucose?" (2b.) "At what intervals do you perform blood glucose checks?" (2c.) "Do you maintain a blood glucose diary?" For those indicating the usage of CGM, the following questions were presented: (3a.) "Do you need help with inserting and calibrating the CGM sensor?" (3b.) "How often do you review your CGM readings?"

When evaluating the degree of adherence, the following criteria were employed: (1.) Adherence to the prescribed method and schedule of glycemic control agreed upon with the treating physician. (2a.) Independent management or a willingness to accept assistance from others in monitoring capillary blood glucose levels. (3a.) Independent management or a willingness to accept assistance from others in inserting and calibrating the CGM sensor. (2b; 3b.) glycemic control at least three times a day before meals and at least three times per week, with assessments performed 1.5−3 h after meals; and (2c.) regular inputs into the blood glucose diary unless the pediatric patient relies on a sensor for CGM. Note: Respondents were classified into two groups: adherent and non-adherent. Patients were categorized as adherent if:

- SMBG was used and the recommended glucose target values between 4 and 10 mmol/L (70.0−180.0 mg/dl) were achieved, with a narrower fasting target range of 4.0−8.0 mmol/L (70.0−144.0 mg/dl), and the respondents selected the response "the frequency of glycemic control at least three times a day before meals, as well as the frequency of blood glucose measurement within 1.5−3 h after meals at least three times a week" [32].
- CGM sensors were used; the CGM metrics (recorded over 14 days) should have a period (%TIR) that is >70.0% between 3.9 and 10.0 mmol/L (70−180 mg/dL) [32].

The evaluation of *dietary adherence* was conducted using multiple-choice closed-response queries to ensure it accurately represents the respondents’ answers and reduces the risk of bias. The questions related to healthy eating habits covering the preceding 30-day period were: (1) How many of the last 30 days have they followed a healthy eating plan? (2) "Did they show a preference for fruits, vegetables, whole grains, and low-fat foods?", as well as (3) controlling carbohydrate intake and mealtime organization (4) "How did they plan their meals, including the frequency of breakfast, lunch, dinner, and snacks?" and the degree of adherence to the diet that participants had reported. It’s important to emphasize that adherence was defined as aligning with the reported diet at a minimum of 90% of the time, equivalent to answers under 1b or 1c; 2b or 2c; 3b or 3c; as well as 4b or 4c.

*Physical activity* was evaluated through answers provided to the subsequent inquiries covering the preceding 30-day period: (1a.) "Did you engage in physical activity (ranging from moderate to vigorous intensity) for 60 minutes or more each day?" (1b.) "Did you participate in sports?" If the response is affirmative, further details are sought: (1c.) "How frequently did you train each week, and what specific sport do you partake in?" Participants were grouped into two categories: those who adhered and those who did not. Patients were labeled as non-adherent if they did not meet the requirement of being physically active (at a moderate to vigorous intensity) for a minimum of 60 minutes every day.

The assessment of *diabetes management during school hours* was carried out by collecting responses to the following questions covering the preceding 30-day period: (1a.) "Did being at school pose challenges for managing your diabetes?" (1b.) If the response was "occasionally", respondents were asked to specify the situations that present difficulties. (2.) "Have you participated in school trips since being diagnosed with diabetes?" As a result, patients were categorized as either pediatric patients facing challenges in diabetes management at school or those who did not encounter such difficulties. If children and adolescents did not participate in school trips after being diagnosed with diabetes and described conditions that made controlling diabetes difficult during their school stay, they were classified as patients facing challenges in diabetes management at school.

Note: When organizing one-day and multi-day school trips in Serbia, the school director (together with the manager of the travel agency) is obliged to designate a pediatrician who is available to the students for the entire duration of the trip, and in case of need.

In a broader sense, pediatric patients were classified as:

- “highly adherent to self-care activities” if they exhibited adherence to three specific components (such as glycemic control, dietary regimen, and physical activity), experienced no significant challenges managing diabetes at school, and maintained an HbA1c level <7.0% (<53.0 mmol/mol); (for example, participants were adherent in glycemic control, dietary regime, and physical activity, without some difficulties in diabetes management in school, and with HbA1c <7.0% (<53.0 mmol/mol).
- Additionally, pediatric patients were labeled as “adherent to self-care activities” if they adhered to two specific components, either with or without some difficulties in diabetes management at school, and if their HbA1c was <7.0% (<53.0 mmol/mol); (for instance, a patient might not strictly adhere to glycemic control and encounter some challenges at school, but still exhibit adherence to the dietary regimen and physical activity, all while maintaining HbA1c levels <7.0% (<53.0 mmol/mol).
- Finally, pediatric patients fell into the category of “low adherence to self-care activities” if they did not adhere to two specific components, faced difficulties in managing diabetes at school, and had an HbA1c level ≥7.0% (≥53.0 mmol/mol); (for instance, a patient might engage in daily physical activity for 60 minutes, but not adhere to glycemic control and dietary guidelines, while also experiencing challenges in school management, all while having an HbA1c level ≥7.0% (≥53.0 mmol/mol).

*Description of the second part of questionnaire*. In the initial task focusing on *insulin therapy*, children and adolescents diagnosed with T1DM were inquired about the following aspects: (1.) "Who typically assumed responsibility for the three components of MDI in the past month?": (1a.) Selecting the dosage—"Did any family member assist you with determining insulin doses?" (1b.) Selecting the injection site—"Did a family member help you decide where to administer insulin?" (1c.) Administering the insulin injection—"Do you administer insulin to yourself?" Alternatively, they were asked: (2.) "Who usually managed the various tasks related to insulin pump therapy in the past month?"

In the subsequent task, which centered on *glycemic control*, children and adolescents living with T1DM were inquired about the typical individuals responsible for various aspects of maintaining glycemic control during the past month: (1a.) Regarding blood glucose checks: "Do you require assistance with setting up the meter and performing fingerstick tests for SMBG?" (1b.) Concerning recording the results: "Do you maintain a record of your blood glucose readings through a blood glucose diary?" (2a.) Likewise, they were queried about CGM: "Who typically undertakes the tasks associated with CGM, such as selecting the sensor placement on the skin, carrying out the insertion and calibration of the CGM sensor?" (2b.) "Who is responsible for removing the CGM sensor?"

Due to the absence of a definitive set of parental behaviors encompassing an "involved parent," the elements of parental involvement in insulin therapy were amalgamated to establish the "insulin-routine score (IRS)". Similarly, the constituent behaviors pertaining to parental participation in glycemic control were consolidated to establish the "glycemic monitoring score (GMS)".

Given that the injection of insulin holds primary significance within insulin administration, either conducting injections or overseeing them formed the central criterion for determining the "IRS". This scoring system encompassed parental involvement levels, ranging from 1 to 4, culminating in a cumulative score spectrum of 3 to 12. Scores were categorized as follows:

- Less than 5 indicated “minimal parental involvement”, characterized by sporadic parental injection or supervision of less than 50% of daily injections, occasionally managing "Correction Bolus."
- Scores between 5 and 10 denoted “moderate parental involvement”, wherein parents were responsible for at least 50% of daily injections, inputting glucose levels and carbohydrate values into the pump, and engaging in "Correction Bolus" management.
- Scores exceeding 10 signified “maximal parental involvement”, where parents independently determined insulin dosages, administered all injections without the child/adolescent’s participation, and managed all tasks pertaining to insulin pump therapy.

The "GMS" gauged the extent to which parents were engaged in ensuring monitoring glucose tasks were executed. This score was determined based on parental participation, ranging from 1 to 4, with a total score range of 2 to 8. Categorized as follows:

- Less than 4 indicated “minimal parental involvement”, encompassing instances where parents reminded the child to log/check results, or supervised the CGM sensor insertion and calibration process, while having knowledge of the outcomes.
- Scores between 4 and 5 denoted “moderate parental involvement”, involving parents who typically checked and logged results, or supervised insertion processes while leaving CGM sensor calibration to the child, with parents retaining awareness of the results.
- Scores exceeding 5 represented “maximal parental involvement”, reflecting situations where parents conducted finger sticks, set up meters, and logged results, or managed the entirety of the process including choosing sensor locations, performing insertions and calibrations of the CGM sensor, as well as sensor removal. Consequently, parents were fully informed about the results.

The level of autonomy exhibited by pediatric patients in managing their illness was established by combining the "IRS" and the "GMS” scores, resulting in a cumulative "diabetes self-management score (DSM score)" with a range of 5 to 20. This composite "DSM score" effectively depicted the degree of independence that pediatric patients displayed in managing their chronic medical condition. Respondents were classified as “independent” if they achieved a score ranging from 5 to 12, while a score within the range of 13 to 20 indicated a “dependent level of self-management”.

### Statistics

For numerical data, various statistical parameters were calculated, including the arithmetic mean, standard deviation, median, trimmed arithmetic mean, absolute deviation of the median, minimum, maximum, range, standard error of the arithmetic mean, distribution skewness, and kurtosis. For categorical (nominal) variables, frequencies and percentages were reported. The normality of the distribution was assessed using the Shapiro−Wilk test, which relied on the skewness and kurtosis values. Differences between two or more groups concerning nominal variables were analyzed using Pearson’s Chi-square test, a method that determines whether a relationship exists between two nominal variables; the degrees of freedom (df) are given in parenthesis after χ2. The effect size for Cramer’s V (V) and its interpretation was done (df1 = 0.10 small, 0.30 medium, 0.50 large; df2 = 0.07 small, 0.21 medium, 0.35 large; df3 = 0.06 small, 0.17 medium, 0.29 large; et all.) [33]. Furthermore, we used the Fisher’s exact test [33]. All statistical analyses were conducted using the R programming language within the R Core Team’s Environment for Statistical Computing [34]. The outcomes of the statistical analysis were presented in textual or tabular formats, with statistical significance indicated by a threshold of p < 0.05.

### Ethical approval

The research was carried out in compliance with the principles outlined in the Declaration of Helsinki and received approval from the Ethics Committees of both the Mother and Child Health Care Institute of Serbia "Dr Vukan Čupić" (IMD: No. 8/21, 21 May 2019) and the University Children’s Hospital in Belgrade (UDK: 017 No. 14/6, 03 January 2020).

## Results

### Sociodemographic characteristics of pediatric patients with T1DM

The sample consisted of 83 children (46.0%) and 99 adolescents (54.0%) diagnosed with T1DM, with 113 (62.0%) being female. The average age of the participants was 12.68 (SD = 3.32) years (more detailed descriptive statistics for this numerical variable are provided in Table 1). No significant variations were observed in terms of age and gender (χ2_(1)_ = 1.5305, p = 0.216). A total of 94 respondents (52.0%) hailed from families with five or more members. Moreover, 176 children and adolescents (97.0%) were enrolled in school full-time. Regarding school performance, 84 participants (46.0%) were rated as excellent, 77 (42.0%) as very good, 19 (10.0%) as good, and 2 (1%) were classified as having sufficient school performance. The research encompassed all regions of Serbia: 106 respondents (58.0%) hailed from the Belgrade region, 26 (14.0%) from Vojvodina, 26 (14.0%) from Southern and eastern Serbia, and 24 (13.0%) from Sumadija and western Serbia.

**Table 1:** Descriptive statistics for numerical variables.

	N	Mean	SD	median	Trimmed	Mad	min	max	range	skewness	kurtosis	SE
Age (years)	182	12.68	3.32	13.00	12.80	3.71	8.00	18.00	14.00	-0.32	-0.71	0.25
Duration of T1DM (years)	182	4.43	3.36	3.00	3.99	2.97	0.00	15.00	15.00	1.02	0.20	0.25
HbA1c%	182	7.88	1.43	7.70	7.72	0.89	4.90	14.70	9.80	1.75	5.03	0.11

Note: Frequency (N); Arithmetic mean (mean); Standard deviation (SD); median

The arithmetic mean reported in the column denoted by “trimmed” was obtained after removing the top and bottom 5% results (outliers)

“Mad” denotes median absolute deviation; “Range” reflects the span between the (min)imal and (max)imal variable values

“Skewness” reflects the distortion or asymmetry of the distribution, whereby a negative (positive) value indicates the greater prevalence of higher (lower) values in the sample

“Kurtosis” is a measure of the combined weight of a distribution’s tails relative to the centre of the distribution, whereby its positive value indicates that the majority of values are clustered around the mean, reducing the variance. Conversely, negative kurtosis indicates a flatter distribution with greater data dispersion

The normal distribution is characterized by skewness and kurtosis approaching zero; (SE) denotes the standard error of the arithmetic mean.

### Clinical characteristics of pediatric patients with T1DM

The mean disease duration since diagnosis T1DM was 4.43 (SD = 3.36) years (more detailed descriptive statistics for this numerical variable are provided in Table 1). Among these, 71 participants (39.0%) had a longstanding condition lasting five years or more. Distribution by age at diabetes onset was as follows: 69 respondents (38.0%) were diagnosed in early childhood (ages 5–9), 70 (38.0%) during ages 10–14, 36 (20.0%) were younger than 5 years at onset, and 7 (4.0%) were diagnosed between ages 15 and 18. Insulin pump therapy was adopted by 106 pediatric patients (58%), while the remaining received MDI. The average HbA1c value was 7.88% (62.6 mmol/mol, with SD = 1.43) (more detailed descriptive statistics for this numerical variable are provided in Table 1). Desired metabolic control, reflected by HbA1c values <7.0% (<53.0 mmol/mol) was observed in 28 pediatric patients (15.39%). Over the past month, 18 pediatric patients (10.0%) experienced severe hypoglycemia, and 8 participants (4.0%) encountered DKA.

### Description of adherence to self-care activities

Our findings revealed that 163 children and adolescents (89.56%) exhibited non-adherence to self-care activities. Descriptive statistics for various clinical variables related to self-care in relation to sociodemographic characteristics are detailed in Table 2. Notably, children and adolescents from the Vojvodina region displayed significantly higher adherence to glycemic control compared to individuals from other regions (χ2_(3)_ = 10.102; p = 0.01; Cramer’s V: 0.236, indicating a moderate effect size; Fisher exact test: p = 0.019, significant). Children reported encountering more frequent difficulties in managing T1DM during their time at school (χ2_(1)_ = 4.562; p = 0.03; Cramer’s V: 0.158, signifying a weak effect size; Fisher exact test: p = 0.037, significant). Additionally, this group of pediatric patients demonstrated a notably lower adherence to the prescribed dietary regimen (χ2_(1)_ = 5.876; p = 0.01; Cramer’s V: 0.181, with a weak effect size; Fisher exact test: p = 0.017, significant).

**Table 2:** Self-care characteristics of pediatric patients regarding different age groups, gender, and region of residence.

Variable	Answer	Age groups		Gender		Region				All patients
		8–12*	13–18	Male	Female	Bg	V*	SES	SWS	
		N (%)	N (%)	N (%)	N (%)	N (%)	N (%)	N (%)	N (%)	N (%)
**Adherence to self-care activities**	**Highly adherent**	3 (3.61)	4 (4.04)	2 (2.90)	5 (4.42)	4 (3.77)	2 (7.69)	1 (3.85)	0 (0.00)	7 (3.85)
	**Adherent**	6 (7.23)	6 (6.06)	4 (5.80)	8 (7.08)	10 (9.43)	1 (3.85)	1 (3.85)	0 (0.00)	12 (6.60)
	**Non-adherent**	74 (89.16)	89 (89.90)	63 (91.30)	100 (88.50)	92 (86.79)	23 (88.46)	24 (92.31)	24 (100.00)	163 (89.56)
Adherence to glycemic control*	**Adherent**	34 (40.96)	40 (40.40)	26 (37.68)	48 (42.48)	41 (36.68)	**17 (65.38)**	6 (23.98)	10 (41.67)	74 (40.66)
	**Non-adherent**	49 (59.04)	59 (59.60)	43 (62.32)	65 (57.52)	65 (61.32)	9 (34.62)	20 (76.92)	14 (58.33)	108 (59.34)
Dietary adherence*	**Adherent**	39 (46.99)	63 (64.95)	39 (56.52)	63 (56.76)	56 (53.33)	17 (68.00)	17 (65.38)	12 (50.00)	102 (56.66)
	**Non-adherent**	**44 (53.01)**	34 (35.05)	30 (43.48)	48 (43.24)	49 (46.67)	8 (32.00)	9 (34.62)	12 (50.00)	78 (43.33)
**Physical activity**	**Adherent**	33 (40.24)	43 (43.43)	25 (36.23)	51 (45.54)	41 (39.05)	12 (46.15)	11 (42.31)	12 (50.00)	76 (41.99)
	**Non-adherent**	49 (59.76)	56 (56.57)	44 (63.77)	61 (54.46)	64 (60.95)	14 (53.85)	15 (57.69)	12 (50.00)	105 (58.01)
Control of T1DM during school*	**No difficulties**	38 (45.78)	61 (61.62)	34 (49.28)	65 (57.52)	57 (53.77)	13 (50.00)	11 (42.31)	18 (75.00)	99 (54.40)
	**Difficulties**	**45 (54.22)**	38 (38.38)	35 (50.72)	48 (42.48)	49 (46.23)	13 (50.00)	15 (57.69)	6 (25.00)	83 (45.60)

Note: The results were interpreted based on Fisher’s exact test; significant results (*p* < 0.05) were highlighted in bold and variables were marked with *

Also, we used Chi-squared Test; significant results (*p* < 0.05) were highlighted in bold, and variables were marked with *

Bg = Belgrade; Vojvodina = V; South & eastern Serbia = SES; Sumadija & western Serbia = SWS.

Differences between adherence to glycemic control regarding regions–Vojvodina χ^2^_(3)_ = 10.102; **p = 0.01**; Cramer’s V: 0.236 (moderate effect size); **Fisher exact test: p = 0.019 (significant).**

Differences between dietary adherence regarding age group χ^2^_(1)_ = 5.876; **p = 0.01**; Cramer’s V: 0.181 (weak effect size); **Fisher exact test: p = 0.017 (significant)**

Differences between difficulties in control T1DM during school stay regarding age χ^2^_(1)_ = 4.562; **p = 0.03**; Cramer’s V: 0.158 (weak effect size); **Fisher exact test: p = 0.037 (significant)**

As outlined in Table 3, the statistical significance was not established within the group that encountered severe hypoglycemia, although there was a tendency for non-adherent individuals to experience it more frequently, and conversely, adherents to experience it less often (χ^2^_(2)_ = 2.328; p = 0.36; Cramer’s V: 0.113, indicating a weak effect size; Fisher exact: p = 0.669 not significant). Similarly, no statistical significance emerged in the comparison between the level of adherence to self-care activities and the incidence of DKA during the preceding month (χ^2^_(2)_ = 0.975; p = 0.76; Cramer’s V: 0.073, indicating weak effect size; Fisher exact: p = 1.000 not significant).

**Table 3:** Self-care characteristics of pediatric patients regarding episodes of severe hypoglycemia and DKA during the previous month.

Variable	Answer	Severe hypoglycemia		DKA		All patients
		No	Yes	No	Yes	
		N (%)	N (%)	N (%)	N (%)	N (%)
**Adherence to self-care activities**	**Highly adherent**	7 (4.27)	0 (0.00)	7 (4.02)	0 (0.00)	7 (3.85)
	**Adherent**	12 (7.32)	0 (0.00)	12 (6.9)	0 (0.00)	12 (6.59)
	**Non-adherent**	145 (88.41)	18 (100.00)	155 (89.08)	8 (100.0)	163 (89.56)

Note: The results were interpreted based on Fisher’s exact test, and there is no difference between the observed groups formed by two variables (all p > 0.05).

Also, we used the Chi-squared Test, and there is no difference between the observed groups formed by two variables.

Differences between adherence to self-care activities regarding episodes of severe hypoglycemia: χ^2^_(2)_ = 2.328; p = 0.36; Cramer’s V: 0.113 indicating a weak effect size; **Fisher exact: p = 0.669, not significant**.

DKA = diabetic ketoacidosis

Differences between adherence to self-care activities regarding episodes of DKA χ^2^_(2)_ = 0.975; p = 0.76; Cramer’s V: 0.073 indicating a weak effect size; **Fisher exact: p = 1.000, not significant.**

### Parental involvement in diabetes management tasks

According to our findings, the occurrence of parental involvement in the “IRS” was notably higher in children (χ^2^_(2)_ = 31.326; p = 0.00; Cramer’s V: 0.415, indicating a moderate effect size; Fisher exact: p = 0.000 (significant)). Other analyzed relationships between parental involvement in the IRS and sociodemographic factors did not yield any statistically significant results. Furthermore, there was no substantial correlation between parental involvement in the “GMS” and sociodemographic characteristics like age, gender, and region of residence, as depicted in Table 4.

**Table 4:** Description of the relationship between level of parental involvement in “IRS” and “GMS”, with different age groups, gender, and region of residence.

Variable	Answer	Age groups		Gender		Region				All patient
		8–12*	13–18	Male	Female	Bg	V*	SES	SWS	
		N (%)	N (%)	N (%)	N (%)	N (%)	N (%)	N (%)	N (%)	N (%)
IRS* parental involvement	**Low**	8 (9.64)	46 (46.46)	16 (23.19)	38 (33.63)	32 (30.19)	9 (34.62)	5 (19.23)	8 (33.33)	54 (29.67)
	Moderate*	**63 (75.90)**	49 (49.49)	44 (63.77)	68 (60.18)	62 (58.49)	16 (61.54)	19 (73.08)	15 (62.50)	112 (61.54)
	**Maximal**	12 (14.46)	4 (4.04)	9 (13.04)	7 (6.19)	12 (11.32)	1 (3.85)	2 (7.69)	1 (3.85)	16 (8.79)
**GMS parental involvement**	**Low**	48 (57.83)	66 (66.67)	45 (65.22)	69 (61.06)	66 (62.26)	13 (50.00)	17 (65.38)	18 (75.00)	114 (62.63)
	**Moderate**	29 (34.94)	32 (32.32)	21 (30.43)	40 (35.40)	36 (33.96)	12 (46.15)	7 (26.92)	6 (25.00)	61 (33.52)
	**Maximal**	6 (7.23)	1 (1.01)	3 (4.35)	4 (3.54)	4 (3.77)	1 (3.85)	2 (7.69)	0 (0.00)	7 (3.85)

Note: The results were interpreted based on Fisher’s exact test; significant results (*p* < 0.05) were highlighted in bold and variables were marked with *

Also, Chi-squared test were calculated; significant results (*p* < 0.05) were highlighted in bold and variables were marked with *

The significant relationship between IRS parental involvement and participants aged 8–12 years χ^2^_(2)_ = 31.326; **p = 0.00**; Cramer’s V: 0.415 indicating a moderate effect size; **Fisher exact: p = 0.000 (significant)**

Bg = Belgrade; Vojvodina = V; South & eastern Serbia = SES; Sumadija & western Serbia = SWS.

IRS = insulin routine score; GMS = glycemic monitoring score

The explored relationships between IRS and clinical characteristics did not yield any statistically significant outcomes. More precisely, the relationship between IRS parental involvement and the occurrence of severe hypoglycemic episodes in the previous month did not yield substantial support in our study ((χ^2^_(2)_ = 6.414; p = 0.04; Cramer’s V: 0.188, indicating a weak effect size; Fisher exact test: p = 0.053 (not significant)). The relationship between IRS parental involvement and the occurrence of DKA episodes in the previous month did not yield substantial support in our study ((χ^2^_(2)_ = 2.082; p = 0.43; Cramer’s V: 0.107 indicating weak effect size; Fisher exact: p = 0.438 (not significant)). The relationship between IRS parental involvement and degree of metabolic control did not yield substantial support in our study ((χ^2^_(6)_ = 3.992; p = 0.68; Cramer’s V: 0.105 indicating weak effect size; Fisher exact: p = 0.631 (not significant)).

Furthermore, the explored relationships between parental involvement in the GMS and clinical characteristics did not yield any statistically significant outcomes. More precisely, the relationship between parental involvement in the GMS and the occurrence of severe hypoglycemic episodes in the previous month did not yield substantial support in our study ((χ^2^_(2)_ = 1.258; p = 0.54; Cramer’s V: 0.083, indicating a weak effect size; Fisher exact test: p = 0.813 (not significant)). The relationship between parental involvement in GMS and the occurrence of DKA episodes in the previous month did not yield substantial support in our study ((χ^2^_(2)_ = 0.361; p = 1.00; Cramer’s V: 0.045 indicating weak effect size; Fisher exact test: p = 1.000 (not significant)). The relationship between parental involvement in GMS and degree of metabolic control did not yield substantial support in our study ((χ^2^_(6)_ = 5.781; p = 0.41; Cramer’s V: 0.126 indicating weak effect size; Fisher exact test: p = 0.402 (not significant)).

Further elaborations on the connection between parental involvement in the “IRS”, “GMS”, and the clinical attributes of pediatric patients are elaborated upon in detail within Table 5.

**Table 5:** Description of the relationship between level of parental involvement in IRS and GMS, with clinical characteristics of pediatric patients with T1DM.

Variable	Answer	Severe hypoglycemia*		DKA		Metabolic control				All patients
		No	Yes*	No	Yes	Ideal	Good	Unstable	Poor	
		N (%)	N (%)	N (%)	N (%)	N (%)	N (%)	N (%)	N (%)	N (%)
**IRS parental involvement**	**Low**	44 (26.83)	10 (55.56)	50 (28.74)	4 (50.00)	0 (0.00)	23 (33.33)	19 (27.54)	12 (30.77)	54 (29.67)
	**Moderate**	105 (64.02)	7 (38.89)	108 (62.07)	4 (50.00)	4 (80.00)	39 (56.52)	46 (66.67)	23 (58.97)	112 (61.54)
	**Maximal**	15 (9.15)	1 (5.56)	16 (9.2)	0 (0.00)	1 (20.00)	7 (10.14)	4 (5.80)	4 (10.26)	16 (8.79)
**GMS parental involvement**	**Low**	101 (61.59)	13 (72.22)	109 (62.64)	5 (62.50)	3 (60.00)	43 (62.32)	42 (60.87)	26 (66.67)	114 (62.64)
	**Moderate**	56 (34.15)	5 (27.78)	58 (33.33)	3 (37.50)	2 (40.00)	22 (31.88)	24 (34.78)	13 (33.33)	61 (33.52)
	**Maximal**	7 (4.27)	0 (0.00)	7 (4.02)	0 (0.00)	0 (0.00)	4 (5.80)	3 (4.35)	0 (0.00)	7 (3.84)

Note: The results were interpreted based on Fisher’s exact test, (all p > 0.05).

The relationships between parental involvement in the IRS and clinical variables (HbA1c level as an indicator of metabolic control, episodes of severe hypoglycemia and DKA) did not yield any statistically significant results.

The relationships between parental involvement in the GMS and clinical variables (HbA1c level as an indicator of metabolic control, episodes of severe hypoglycemia, and DKA) did not yield any statistically significant results.

Also, the Chi-squared test was calculated, and the significant relationship between IRS parental involvement and the occurrence of severe hypoglycemia was not fully determined because the p-value is borderline (p = 0.04).

IRS = insulin routine score; GMS = glycemic monitoring score; DKA = diabetic ketoacidosis

Ideal metabolic control with HbA1c% <5.7% (<38.8 mmol/mol)

good metabolic control with HbA1c% in the 5.7−6.9% range (corresponding to 38.8−51.9 mmol/mol)

HbA1c% in the 7.0−8.5% range (53.0−69.4 mmol/mol) signifying unstable metabolic control, and

HbA1c% > 8.5% (>69.4 mmol/mol) indicating poor metabolic control

### Relationship between diabetes self-management and sociodemographic and clinical characteristics

Illustrated in Table 6, a majority of the study participants, totaling 142 individuals (78.0%), affirmed their autonomy in managing their diabetes. However, children notably displayed a greater reliance on parental involvement in handling tasks related to disease management (χ^2^_(1)_ = 14.95; p = 0.00; Cramer’s V: 0.287, signifying a moderate effect size; Fisher exact: p = 0.000 (significant)). Other explored relationships between DSM and sociodemographic characteristics did not yield any statistically significant outcomes.

**Table 6:** Description of the relationship between diabetes self-management with different age groups, gender, region of residence, and clinical characteristics.

Variable	Answer	Age groups		Gender		Region				All patients
		8–12*	13–18	Male	Female	Bg	V*	SES	SWS	
		N (%)	N (%)	N (%)	N (%)	N (%)	N (%)	N (%)	N (%)	N (%)
**Diabetes self-management**	**Independent of parental involvement**	54 (65.06)	88 (88.89)	49 (71.01)	93 (82.30)	79 (74.53)	23 (88.46)	19 (73.08)	21 (87.50)	142 (78.02)
	Dependent of parental involvement*	**29 (34.94)**	11 (11.11)	20 (28.99)	20 (17.70)	27 (25.47)	3 (11.54)	7 (26.92)	3 (12.50)	40 (21.98)
		**Severe hypoglycemia**		**DKA**		**Metabolic control**				**All patients**
		**No**	Yes*	**No**	**Yes**	**Ideal**	**Good**	**Un-stable**	**Poor**	
		**N (%)**	**N (%)**	**N (%)**	**N (%)**	**N (%)**	**N (%)**	**N (%)**	**N (%)**	**N (%)**
**Diabetes self-management**	**Independent of parental involvement**	126 (76.83)	16 (88.89)	134 (77.01)	8 (100.0)	4 (80.00)	54 (78.26)	53 (76.81)	31 (79.49)	142 (78.02)
	**Dependent of parental involvement**	38 (23.17)	2 (11.11)	40 (22.99)	0 (0.00)	1 (20.00)	15 (21.74)	16 (23.19)	8 (20.51)	40 (21.98)

Note: The results were interpreted based on Fisher’s exact test; significant results (*p* < 0.05) were highlighted in bold and variables were marked with *

The significant relationship between dependent of parental involvement in disease management tasks and pediatric patients aged 8–12; (χ^2^_(1)_ = 14.95; **p = 0.00**; Cramer’s V: 0.287, signifying a moderate effect size; **Fisher exact: p = 0.000 (significant)**

Also, Chi-squared test were calculated; significant results (*p* < 0.05) were highlighted in bold and variables were marked with *

Bg = Belgrade; Vojvodina = V; South & eastern Serbia = SES; Sumadija & western Serbia = SWS.

DKA = diabetic ketoacidosis

Ideal metabolic control with HbA1c% <5.7% (<38.8 mmol/mol)

good metabolic control with HbA1c% in the 5.7−6.9% range (corresponding to 38.8−51.9 mmol/mol)

HbA1c% in the 7.0−8.5% range (53.0−69.4 mmol/mol) signifying unstable metabolic control, and

HbA1c% > 8.5% (>69.4 mmol/mol) indicating poor metabolic control

The link between self-management independence and factors such as metabolic control (χ^2^_(3)_ = 1.508; p = 0.69; Cramer’s V: 0.091, signifying a weak effect size; Fisher exact: p = 0.733 (not significant)), as well as the incidence of severe hypoglycemia and DKA during the previous month, did not yield substantial support in our study χ^2^_(1)_ = 1.376; p = 0.36; Cramer’s V: 0.087, signifying a weak effect size; Fisher exact: p = 0.370 (not significant)), and χ^2^_(1)_ = 2.357; p = 0.21; Cramer’s V: 0.114, signifying a weak effect size; Fisher exact: p = 0.203 (not significant)).

## Discussion

Based on the results obtained in this study, adherence to self-care activities in children and adolescents with T1DM in Serbia is suboptimal. Estimated differences in adherence to self-care activities in pediatric T1DM patients regarding sociodemographic (age, gender, or region of residence) or clinical characteristics (episodes of severe hypoglycemia or DKA in the previous month) did not reach statistical significance, confirming the first hypothesis. The second hypothesis was mostly confirmed. More precisely, the estimated relationship between parental involvement in diabetes self-management and sociodemographic (gender or region of residence) or clinical characteristics (HbA1c level as an indicator of metabolic control, particularly with episodes of severe hypoglycemia or DKA in the previous month) did not reach statistical significance. However, parental involvement in diabetes self-management is an important predictor of optimal self-care behavior, particularly in the context of insulin therapy in children aged 8 to 12, as discussed below.

Otherwise, we found that children and adolescents with T1DM from the Vojvodina region demonstrated higher adherence to glycemic control than their counterparts from other regions. This might be attributed to Vojvodina having the highest number of newly diagnosed pediatric T1DM cases in 2021. The context of diabetes-specific functioning among parents of newly diagnosed children with T1DM could explain this, with parents likely monitoring their children’s glucose levels more frequently due to concerns about hypoglycemia [35]. However, fear of hypoglycemia is common among parents of children and adolescents with T1DM, and it has been linked to maladaptive behaviors to avoid low blood glucose levels [36].

It’s worth noting that children aged 8 to 12 faced more challenges in managing T1DM during their time in school, which concurs with a Serbian study from 2016 [37]. Furthermore, this subgroup of pediatric patients showed less adherence to the prescribed dietary regimen, a somewhat surprising finding, suggesting that a comprehensive understanding of their nutritional habits is essential. Also, nutritional education for children and families in such cases is imperative, as the correlation between glycemic control and dietary adherence has been established [38].

In this comparative cross-sectional and correlational study, a substantial proportion of the research participants asserted their autonomy in diabetes self-management. However, a very high percentage of pediatric patients with T1DM did not adhere to self-care activities, a finding commonly observed among adolescents [8, 9]. Therefore, it is vital to understand how parents are involved in the management of their child’s diabetes. Based on the results obtained in this study, pediatric patients with T1DM in Serbia gradually acquire the skills to independently manage their condition, often starting from childhood. Initial self-care steps often encompass tasks like glucose monitoring and insulin injections. However, important components of self-care include following a healthy diet, regular physical activity, monitoring blood glucose, adherence to insulin therapy, and having healthy coping and problem-solving skills related to diabetes self-management [39]. Multiple authors provided evidence that points to an inverse association between adherence to self-care activities and HbA1c level as an indicator of metabolic control [40, 41]. Nurses should talk particularly to adolescents with T1DM regarding the desired metabolic control and activities in which they participate.

Consequently, it’s unsurprising that 84.62% of pediatric patients with T1DM exhibited unsatisfactory metabolic control, marked by HbA1c levels exceeding 7.0% (53.0 mmol/mol). This aligns with similar studies [9, 11, 16, 21, 42].

Our findings indicated that non-adherents to self-care experienced episodes of severe hypoglycemia more frequently, and vice versa, although statistical significance wasn’t fully established (p = 0.06). This could be due to our recording of severe hypoglycemia episodes over the previous month. Moreover, 4% of pediatric patients experienced DKA in the past month, with two cases involving intentional insulin dosage manipulation. One female adolescent deliberately underdosed insulin for weight loss, while another engaged in self-harm to attract attention. These occurrences are consistent with known reasons for insulin manipulation [43]. Parental or guardian involvement in diabetes management remains crucial during the high-risk transition to adulthood, particularly for female adolescents, as several studies have confirmed that young females are more likely to engage in nonsuicidal self-injury than males [44, 45]. Additionally, adherence to self-care activities holds paramount importance for pediatric T1DM patients, as it serves as a predictor for both short-term and long-term health outcomes. Al Alshaikh and Doherty [46] in a review paper (from 2023. year) point out that DKA may associated with increased rates of self-injurious behaviours. A systematic approach for assessing psychiatric disorders in patients manipulating insulin therapy is strongly advocated.

Furthermore, the present study unveiled that age has emerged as a sociodemographic factor significantly tied to parental involvement. Despite the reliance on self-reported data, these findings aligned with earlier research literature [20, 41, 47–50]. The present study did not establish a substantial link between parental involvement in the “IRS” and the occurrence of a severe hypoglycemic episode in the preceding month. Further exploration is warranted to gain a deeper comprehension of this relationship. A known factor is that numerous children struggle to recognize and effectively communicate the symptoms of low blood glucose [51, 52]. Consequently, there’s a pressing need to initiate re-education efforts aimed at both parents and their children, fostering a more responsible approach to diabetes management.

The present study did not establish a substantial link between participants’ self-management independence in handling their disease and their metabolic control. Nevertheless, prior assessments emphasize the significance of the extent of parental engagement in self-disease control. It’s been observed that when adolescents perceive their parents to be overly involved in diabetes care, and perceive parental control as excessive, it can have an adverse impact on metabolic control [9, 53, 54]. Interestingly, our findings contrast this, revealing that there was no substantial inverse correlation between parental involvement in managing disease tasks and HbA1c levels, which serve as an indicator of metabolic control.

Our study highlights that childhood poses specific challenges for self-care, necessitating parental involvement, particularly in the context of insulin therapy, a finding that aligns with conclusions drawn from several studies [20, 43–50].

Compliance with a nutritious diet and engagement in physical activity constitute essential facets of diabetes self-management [21, 47, 55, 56]. Substantial enhancement in metabolic control and a lowered likelihood of enduring long-term complications can be achieved by the majority of pediatric T1DM patients through improvements in dietary choices, consumption patterns, and physical activity levels. Notably, diets like the Mediterranean diet (MedDiet), which emphasizes whole grains, monounsaturated fats, plant-based foods, and reduced intake of processed meats, have been recommended for long-term health benefits and to mitigate cardiovascular risks [47]. Nonetheless, it’s worth mentioning that adherence to dietary prescriptions was subpar among the younger cohort in our study (aged 8–12). This could potentially be attributed to inadequate vegetable consumption and heightened challenges in managing T1DM during school hours. This observation could be attributed to their penchant for indulging in unhealthy snacks with their peers during school breaks, which resonates with findings reported in other studies [55, 56].

Within our study, a prevalent trend of non-adherence to physical activity was evident among respondents, and the assessment of sociodemographic variables did not yield statistically significant differences. The underlying causes for this insufficiency in physical activity remain enigmatic. One plausible explanation could be the timeframe of our research, which coincided with the initial 20 months of the COVID-19 pandemic. During this period, children and adolescents with T1DM faced alterations in their daily activity routines, often failing to attain a threshold of moderate or vigorous physical activity. This effect was particularly pronounced between March 18 and May 7, 2020, when temporary “lockdown” phases were enforced in Serbia as a precautionary measure for public health. This viewpoint is congruent with the findings of Shah et al. (2022) and Telford et al. (2021) [57, 58]. However, it is important to note that physical activity has proven to exert positive effects on the cardiometabolic health of pediatric T1DM patients, emphasizing the need for robust encouragement in this regard [59].

The heightened incidence of pediatric T1DM cases in Serbia places a substantial burden on the healthcare system, notably on tertiary institutions such as the Mother and Child Health Care Institute of Serbia “Dr Vukan Čupić” and the University Children’s Hospital in Belgrade, as well as secondary facilities like the specialized “Bukovička Banja” hospital in Arandjelovac. The latter serves as a hub for rehabilitation and comprehensive self-care education for children, adolescents, and their parents across all regions of Serbia. To further enhance the effectiveness of diabetes management education, we advocate for the establishment of a home care program akin to the model adopted in France (specifically Paris and Marseille) [60]. This approach has the potential to provide valuable educational resources and support to parents and their children in mastering self-care activities for diabetes management. Health Centers in Serbia feature departments dedicated to community health nursing. Working with the family, and thus with the parents of chronically ill pediatric patients is one of the basic activities of community health nursing in Serbia. We believe that work with pediatric patients with T1DM and their parents could be intensified and improved in home visiting/care—by better coordination of the activities of the community health nursing and micro, mezzo, and macro levels of the health care system.

### Study limitations

Firstly, due to the absence of translated and validated questionnaires in the Serbian language addressing the variables of interest in this study, the study’s lead researcher and co-authors developed the self-report questionnaires utilized in the research. Subsequently, the questionnaires employed to gauge adherence to self-care activities and the extent of parental involvement in disease self-management relied on self-report measures, inherently susceptible to social and recall biases. Owing to the sample was not chosen randomly, the accuracy of data collected can be compromised.

### Relevance to clinical practice

- This study highlights the need for interventions aimed at improving competence in self-care activities in children and adolescents with T1DM in Serbia.
- Healthcare professionals should focus on providing structured educational interventions for pediatric patients with T1DM and their families regarding self-care activities while acknowledging the positive attitudes and healthy habits they already possess.
- These basic results on self-care activities and level of parental involvement in disease self-management can be used to create Serbian versions of specific questionnaires for the assessment of various preferences that shape everyday life and the process of assuming independence in the self-management of diabetes in children and adolescents.

## Conclusion

Drawing from the compelling statistically significant outcomes yielded by our analyses, it becomes apparent that the majority of children and adolescents grappling with T1DM characterize themselves as not adhering to self-care routines. Additionally, our exploration failed to reveal any noteworthy sociodemographic distinctions (age, gender, or region of residence) or clinical variations (instances of severe hypoglycemia or DKA episodes within the preceding month) in terms of adhering to self-care practices.

Relying on the self-reported data provided by the participants, it can be inferred that pediatric T1DM patients tend to display a significant degree of autonomy in managing their condition. However, children, in particular, exhibited a marked reliance on parental involvement when it came to the execution of disease management tasks, especially those involving insulin administration.

These outcomes hold the potential to enrich our comprehension of the intricate interplay between self-care behaviors and the level of parental engagement in diabetes self-management among pediatric T1DM patients in Serbia. In light of this, it’s prudent to consider delving further into this realm through qualitative research avenues.

## Supporting information

S1 Appendix (DOCX)
