# Supplementary material for: Self-care activities in pediatric patients with type 1 diabetes mellitus
Source: PLoS One. 2024 Mar 5;19(3):e0300055. doi: 10.1371/journal.pone.0300055 (PMC10914259; doi:10.1371/journal.pone.0300055)
Supplement: S1 Appendix — (DOCX) [file pone.0300055.s001.docx]

**APPENDIX**

| **ADHERENCE TO SELF-CARE ACTIVITIES**  The questions below ask you about your diabetes self-care activities  during the past 30 days |
| --- |
| **I Adherence to glycemic control (Remember the previous month)** |
| 1. **How did you manage your glucose levels?** 2. **Self-monitoring of blood glucose levels (SMBG)**   a_1_) Did you require assistance when measuring capillary blood glucose?  a_2_) At what intervals did you perform blood glucose checks?  a_c_) Did you maintain a blood glucose diary?   1. **Continous glucose monitoring (CGM)**   b_1_) Did you need help with inserting and calibrating the CGM sensor?  b_2_) How often did you review your CGM readings |
| **II Dietary adherence (Remember the previous month)** |
| 1. **How many of the last 30 days have you followed a healthy eating plan?** 2. I have not followed a healthy eating plan during the past 30 days at all 3. I have followed a healthy eating plan for the past 30 days 4. I haven't followed a healthy eating plan for two days in the past month 5. I haven't followed a healthy eating plan for more than three days in the past month. 6. **How well did you follow the diet regimen?**   a) Fruits, vegetables, whole grains, and low-fat foods are not among my favorites. I usually don't eat that food.  b) I enjoy eating fruits, vegetables, whole grains, and low-fat foods. I have not committed a single offense in the previous month.  c) I enjoy eating fruits, vegetables, whole grains, and low-fat foods. However, I committed an offense during the previous month for two days.  d) I know I should eat fruits, vegetables, whole grains, and low-fat foods. However, I committed an offense more than three times in the past month.   1. **Did you control your carbohydrate intake?** 2. No, I did not 3. Yes, I did control carbohydrate intake for all three main meals and snacks (morning, afternoon, and evening snacks) each day 4. I neglected to control my carbohydrate intake no more than two times in the previous month when I craved my favorite foods 5. I know I need to control carbohydrate intake, but I made the mistake of not doing so more than three times in the last month. 6. **How did you plan your meals, including the frequency of breakfast, lunch, dinner, and snacks?**   a) I never planned when I would eat or what it would be.  b) In agreement with the parents, I made a plan for all meals, including the frequency of breakfast, lunch, dinner, and snacks. I complied with everything.  c) In agreement with my parents, I made a plan for the main meals and snacks, but I was not consistent; I did not follow the daily dietary prescription for two days.  d) I tried to agree with my parents about taking main meals and snacks, but I had a changeable appetite and more than three times did not stick to the agreement. |
| **III Physical activity (Remember the previous month)** |
| 1. **Did you engage in physical activity (ranging from moderate to vigorous intensity) for 60 minutes or more each day?**   a) Yes  b) No   1. **Did you participate in sports?**   a) Yes  a_1_) How frequently did you train each week, and what specific sport do you partake in?  b) No |
| **IV Challenges encountered while managing diabetes at school (Remember the previous month)** |
| 1. **Did being at school pose challenges for managing your diabetes?** 2. No 3. Occasionally   b_1_) Specify the situations that presented difficulties |
| **V School trips** |
| 1. **Have you participated in school trips since being diagnosed with diabetes?** |
| **PARENTAL INVOLVEMENT IN DIABETES**  **SELF-MANAGEMENT**  The questions below ask you about parental involvement in diabetes management |
| **I Administering insulin therapy (Remember the previous month)** |
| 1. **Who typically assumed responsibility for the three components of MDI in the past month**? 2. Selecting the dosage - Did any family member assist you with determining insulin doses? 3. Selecting the injection site - Did a family member help you decide where to administer insulin? 4. Administering the insulin injection - Did you administer insulin to yourself? 5. **Who usually managed the various tasks related to insulin pump therapy in the past month?** |
| **II Assessment of independence in glycemic control**  **(Remember the previous month)** |
| 1. **Regarding blood glucose checks** **and recording the results:**   1a) Did you require assistance with setting up the meter and performing fingerstick tests for SMBG?  1b) Did you maintain a record of your blood glucose readings through a blood glucose diary?   1. **Regarding CGM:**   2a) Who typically undertakes the tasks associated with CGM, such as selecting the sensor placement on the skin, carrying out the insertion and calibration of the CGM sensor?  2b) Who was responsible for removing the CGM sensor? |
